# Schools as potential vaccination venue for vaccines outside regular EPI schedule: results from a school census in Pakistan

**DOI:** 10.1186/1756-0500-5-6

**Published:** 2012-01-06

**Authors:** Sajid Bashir Soofi, Inam-ul Haq, M Imran Khan, Muhammad Bilal Siddiqui, Mushtaq Mirani, Rehman Tahir, Imtiaz Hussain, Mahesh K Puri, Zamir Hussain Suhag, Asif R Khowaja, Abdul Razzaq Lasi, John D Clemens, Michael Favorov, R Leon Ochiai, Zulfiqar A Bhutta

**Affiliations:** 1 Department of Pediatrics and Child Health, Aga Khan University, Karachi, Pakistan; 2 Translational Research Division, International Vaccine Institute, Seoul, Republic of Korea

**Keywords:** Vaccine, typhoid fever, developing country, infectious disease, health education

## Abstract

**Background:**

Vaccines are the most effective public health intervention. Expanded Program on Immunization (EPI) provides routine vaccination in developing countries. However, vaccines that cannot be given in EPI schedule such as typhoid fever vaccine need alternative venues. In areas where school enrolment is high, schools provide a cost effective opportunity for vaccination. Prior to start of a school-based typhoid vaccination program, interviews were conducted with staff of educational institutions in two townships of Karachi, Pakistan to collect baseline information about the school system and to plan a typhoid vaccination program. Data collection teams administered a structured questionnaire to all schools in the two townships. The administrative staff was requested information on school fee, class enrolment, past history of involvement and willingness of parents to participate in a vaccination campaign.

**Results:**

A total of 304,836 students were enrolled in 1,096 public, private, and religious schools (Madrasahs) of the two towns. Five percent of schools refused to participate in the school census. Twenty-five percent of schools had a total enrolment of less than 100 students whereas 3% had more than 1,000 students. Health education programs were available in less than 8% of public schools, 17% of private schools, and 14% of Madrasahs. One-quarter of public schools, 41% of private schools, and 43% of Madrasahs had previously participated in a school-based vaccination campaign. The most common vaccination campaign in which schools participated was Polio eradication program. Cost of the vaccine, side effects, and parents' lack of information were highlighted as important limiting factors by school administration for school-based immunization programs. Permission from parents, appropriateness of vaccine-related information, and involvement of teachers were considered as important factors to improve participation.

**Conclusions:**

Health education programs are not part of the regular school curriculum in developing countries including Pakistan. Many schools in the targeted townships participated in immunization activities but they were not carried out regularly. In the wake of low immunization coverage in Pakistan, schools can be used as a potential venue not only for non-EPI vaccines, but for a catch up vaccination of routine vaccines.

## Background

Vaccination is considered to be the most cost-effective public health tool in preventing infectious diseases responsible for child mortality in developed and developing countries [1]. Immunization programs have led to global eradication of smallpox, elimination of measles and poliomyelitis from certain regions of the world, and substantial reductions in morbidity and mortality attributed to diphtheria, tetanus, pertussis, pneumonia and infectious diarrhea [2]. Such successes have resulted due to the use of safe and effective vaccines against these diseases, in addition to the control of important risk factors. However, the challenge of achieving continued high coverage rates for routine vaccines in developing countries persists [3]. It is estimated that 1.4 million child deaths occur each year due to vaccine-preventable diseases in developing countries [4-6].

The Expanded Program on Immunization (EPI) was introduced in 1974 with the aim of reducing vaccine-preventable deaths in children in developing countries. The coverage rate for DTP3 under EPI increased to almost 75% by the 1990s globally and to 82% in 2009 [7,8]. However, new vaccines that have become available are yet to be introduced in routine vaccination programs in developing countries where cost is a major factor in limiting the use of new vaccines. In addition, the existing health delivery system in developing countries may not be able to bear the load of storage and supply of new-generation vaccines [9]. In areas of high school enrolment, schools provide an opportunity for a catch-up vaccination for routine vaccines such as measles and routine vaccination for vaccines that cannot be administered under EPI such as typhoid. In most developed countries, immunization record is a requirement for school enrolment, and additionally, schools have been used as a platform for immunization for measles, Japanese Encephalitis, influenza, and HPV. Such a strategy for developing countries has been under discussion and several assessments have been made by the World Health Organization (WHO) [10]. Similarly, the Global Immunization Vision and Strategy (GIVS) outlines the need for expansion of "vaccination beyond the traditional target group of infants" [11]. Health education in school settings is increasing in the developing countries, but immunization programs at schools have been mostly limited to campaigns and not in routine [12]. Only few developing countries have implemented routine school immunization programs.

Pakistan has a high incidence of typhoid fever (> 100/100,000 population per year), a systematic illness caused by the bacteria *Salmonella *Typhi [13]. Ninety-seven percent of culture-proven typhoid cases were identified in children 2-12 years of age [14]. However, currently available typhoid vaccines are recommended only for children older than 2 years and hence, typhoid vaccination cannot be incorporated into the routine EPI program. A targeted immunization program in urban areas that uses schools as a platform may be a cost-effective strategy considering that typhoid incidence is high in Pakistan's urban settings, immunization coverage is sustained with previous experience in measles catch up campaigns and polio vaccination campaigns at school settings, and that school enrolment rate is higher in urban settings compared to rural settings [5,15].

The old-generation killed whole cell typhoid vaccines were used in schools in developing countries, including Pakistan, in the 1970s. However, due to high rates of adverse events, the vaccination program was discontinued. But the accumulation of recent evidence of the burden of typhoid from population-based studies and the availability of the Vi polysaccharide vaccine in Pakistan have resulted in growing interest from the public health sector for wider use of typhoid vaccines in children [14,16,17]. As part of the Vi-based Vaccines for Asia (VIVA) Initiative,^a ^which aims to accelerate the adoption of Vi polysaccharide vaccines into developing countries of Asia, a pilot school-based immunization program in Karachi, Pakistan was developed. In preparation for the program, we conducted a census of schools in the study setting to collect information on total enrolment, school location and willingness of school administration to participate in a school-based typhoid vaccination program, and to identify any factors that will increase their participation in such a program. We assessed to find out if there were any social and health service delivery determinants in increasing access to immunization services in Pakistan. In this paper, we present the findings from the school census and implications for a school-based vaccination program, particularly for typhoid fever, in Pakistan.

## Methods

### Study site

The school-based Vi polysaccharide vaccination program took place in Karachi, Pakistan. Karachi, with a total population of approximately 16 M (based on 1998 the population census was 9,856,318, with growth rate of 3.56% per annum), is administratively divided into 17 townships among which Gulshan-e-Iqbal and Jamshed towns were selected to pilot the initial typhoid vaccination campaign [18]. Gulshan-e-Iqbal town is geographically divided into 13 Union Councils (UCs) and has a total population of 1.2 M, while Jamshed town has 13 UCs and has a total population of 730,000 individuals [19]. These two townships have a mix of public, private, and religious schools with representation from various socio-demographic strata, which could provide evidence for future expansion of school-based vaccination campaign in other parts of the city and the country. These towns also have the highest number of culture-proven typhoid fever cases as reported through Aga Khan University's laboratory network system (Unpublished data from the Aga Khan University Hospital Clinical Laboratory).

### Study design

A census of all educational institutions was conducted in the targeted townships. The purpose of inclusion of all institutions was to identify and obtain a comprehensive list of educational establishments where children aged 5-15 years were enrolled. This was considered necessarily since the educational establishments in Pakistan change very frequently from opening, closing, merger and division, and that the government statistics do not capture these changes regularly. In addition, the school census was aimed to provide information on the approximate number of target population for vaccination activity in these educational institutions.

### School system

Three types of educational institutions exist in these townships, as well as in Pakistan: government-operated public schools, private schools that charge a tuition fee, and religious schools that may or may not charge a fee. Most religious schools provide formal education; however, some only teach the Quran and basic Islamic teachings.

Public schools are established under the law of the province and are regulated by the provincial Department of Education. A majority of these schools provide education at no cost or charge a very nominal annual fee. These schools are monitored by government authorities at city, provincial, and federal levels.

Private schools in Pakistan are established, controlled, and run by individuals, an organization, or an autonomous body. Such schools are supported by an endowment and tuition fees, and are monitored by the Provincial Ministry of Education through the Directorate of Private School Association.

Religious schools or Madrasahs are educational institutions that provide religious education with or without formal education. These fall under two categories: those controlled and monitored by an Imam of a mosque, any mosque authority, or any religious organization, and which mainly provide religious education, as well as boarding and lodging at no fee to students; and those that may also provide formal school education along with religious education. Such institutions are supported by an endowment and monthly tuition fees from students. They are usually affiliated with school examination boards and also award certificates to graduating students. Financial support for these institutions may also come from the community, private sector, and politically affiliated parties. The schools' administrative procedures are guided by a committee headed by the Imam of the mosque.

### Collection and analysis

Health and education authorities at city and provincial levels were contacted through seminars and meetings before the start of field activity in order to provide information on the objectives of the school census, as well as the upcoming vaccination program. Memoranda of Understanding (MoU) were signed among Aga Khan University, Ministries of Education and Health, and Town Administrators regarding collaboration on the upcoming activities. Standard Operating Procedures for the field activity and training materials were also developed prior to the census.

A mapping activity was carried out prior to the census to guide the data collection teams for the school visits. Twenty four people were hired and trained on data collection procedures, project scope, and objectives. Extensive training and mock sessions were also conducted on Personal Digital Assistants (PDA) use. There were three groups, with each group led by a field supervisor, consisting of four teams of two people that visited the schools to collect information.

Information about the schools was collected by visiting each school in the study setting. The data collection teams referred to a list of schools provided by the provincial Ministry of Education and Private School Association. The teams also visited each street of the two townships to ensure that all schools were captured during the data collection activity. Prior to data collection visit, the census teams met with a representative of the educational institution and consent was taken for collection of information related to schools. Once the staff member agreed to provide information, the data collection teams administered a structured questionnaire through PDA to the head of each school or other assigned representative. Information on school fee, enrollment by class, past history of involvement in any vaccination campaign, willingness of parents to participate in a vaccination campaign, and additional information related to vaccines, vaccination, and school health practices were collected.

In addition, respondents were asked about difficulties that may be encountered during initiation of a school-based immunization program in Karachi. The respondents were also asked to comment on factors that may be used effectively to promote vaccine uptake among school-aged children in their respective schools. For both questions, they were asked to choose three factors from a list of five and rank them in order of importance.

Quality check and assurance procedures were adopted to ensure quality of the data. An independent monitoring team visited several schools that were randomly sampled from the school list (5% of schools on the list) to verify the census teams' visits to the schools and the appropriateness and accuracy of information.

The electronic version of the data collection questionnaire was developed using Visual Basic of Microsoft Visual Studio 2008 (Microsoft, WA, US). Data were directly saved as text file in PDA and later uploaded as a database file format using Microsoft FoxPro 7.0 (Microsoft, WA, US). Programs were written in FoxPro to identify and clean up range errors, data inconsistency, or logical errors. Reviews of error listings were performed by the field teams and resolved on real-time basis. Corrections were provided to data management personnel to incorporate in the electronic version of the data set. Progress reports were generated on a daily basis by the data management team and shared with field supervisors.

We performed descriptive analysis to calculate the distribution of various variables of interest. For categorical variables proportions and for continuous variables means along with their respective 95% confidence interval were calculated. Stratified analysis was performed to check the distribution between type of schools i.e. public, private and Madrasah. Similarly, within private schools monthly schools fee was used as another variable for stratified analysis. Inferential statistics were not used as this was beyond the scope of this paper. All analysis was performed in statistical software STATA version 11 (StataCorp LP, TX, US).

### Ethical clearance

The school census, which was a part of the pilot typhoid Vi polysaccharide vaccine introduction project, was approved by the Ethical Review Committee (ERC) at Aga Khan University and by the Institutional Review Board (IRB) at the International Vaccine Institute (IVI).

## Results and Discussion

In 39 days with a total of 24 data collection personnel and three field supervisors, we were able to collect data from 1,096 schools in two towns of Karachi. Fifty-eight (5%) schools refused to provide data to our field teams. The reasons for refusals were: absence of an authorized person to provide data at the time of the visit, uncertainty by school administration about the vaccine type and need for its use at the school level, and previous unsatisfactory experience with vaccination or health-related activity. In total, 304,836 students were enrolled in public, private, and religious schools in Gulshan-e-Iqbal and Jamshed towns. Twenty-five percent (276) of all schools had an enrolment of less than 100 students whereas 3% (36) had an enrolment of more than or equal to 1,000 children (Table 1).

**Table 1:** Basic characteristics of schools in two townships of Karachi, Pakistan

	Gulshan		Jamshed		Total	
	
	N	%	n	%	n	%
**Total number of schools**	543		553		1,096	

**Total number of students**	153,472		151,364		304,836	

**Total School Enrolment**						

<100	131	24	145	26	276	25

100- <500	334	62	330	60	664	61

500- <1,000	59	11	61	11	120	11

> = 1,000	19	3	17	3	36	3

**School type**^†^						

Public	53	10	172	31	225	21

Private	421	78	344	62	765	70

Madrasah	66	12	34	6	100	9

**Medium of education**^†^						

English	418	77	338	61	756	69

Urdu	55	10	183	33	238	22

Sindhi	12	2	3	1	15	1

Arabic	55	10	28	5	83	8

Minimum monthly school tuition fee (mean/median) in USD*						

Public	0/0		0/0		0/0	

Private	10.1/6.3		7.7/4.4		9.1/5.0	

Madrasah	1/0		1.8/1.3		1.1/0.4	

**Availability of welfare system at school**^$^						

Public	19/50	38	108/172	63	127/222	57

Private	287/413	69	254/341	74	541/755	72

Madrasah	46/64	72	20/34	59	66/98	67

**Availability of health education at school**^$^						

Public	4/53	8	15/172	9	19/225	8

Private	60/414	14	67/343	19	127/757	17

Madrasah	12/66	18	2/34	6	14/100	14

**Availability of medical care at school**^$^						

Public	38/53	72	117/172	68	155/225	69

Private	383/414	93	306/341	90	689/755	91

Madrasah	46/66	70	14/34	41	60/100	60

^†^percentages are mentioned for responses where participants responded to the questions

^$ ^Missing information, denominators are given along with positive responses

*1USD = 80PKR

The average minimum school fee for schools charging a fee ranged from 615 to 821 rupees^b ^(7.60 to 10.18 USD) per month. Jamshed town had a lower median minimum school fee of 350 rupees (4.30 USD) per month. A welfare system to support the educational expenses of deserving students was available in 56% of public schools, 71% of private schools, and 66% of Madrasahs. Approximately 8% of public schools, 17% of private schools, and 14% of Madrasahs provided some form of health education (Table 1).

One-quarter of public schools, 41% of private schools, and 23% of Madrasahs previously participated in a vaccination campaign that used the school as a venue. The majority (275/1,090) of these school-based campaigns were for polio vaccination, which was carried out under Pakistan's polio eradication program. Ninety-five schools provided vaccines that were not included in the government immunization programs (e.g., EPI, polio eradication program, and measles second chance program). These vaccines were typhoid (provided in 5 schools), varicella (3 schools), and hepatitis B (89 schools). Many of these vaccines were sold at a price, especially in the private schools (Table 2).

**Table 2:** Vaccination experience of schools in two townships of Karachi, Pakistan

	Gulshan		Jamshed		Total	
	**n**	**%**^†^	**N**	**%**^†^	**n**	**%**^†^

**History of prior vaccination campaign**^†^						

Public	11/53	21	46/171	27	57/224	25

Private	157/413	38	152/338	45	309/751	41

Madrasah	17/66	26	6/34	18	23/100	23

**Price of vaccine (mean/median/max) in USD^$^**						

Public	0/0/0		0/0/0		0/0/0	

Private	1.6/0/25		2.1/1.8/11.3		1.7/1.1/25	

Madrasah*	0.2/0/0.63		0.4/0/3.6		0.9/0/3.6	

†The percentages correspond to number of school that provided information of these variables, as not all schools provided information on all variables. Therefore denominators are different for some variables ^$^1USD = 80PKR *single observation

Vaccine price was frequently cited (59% of respondents) as first choice as a barrier to vaccination of children by parents in schools in Gulshan-e-Iqbal town. In Jamshed town, the proportion was even higher (71%). Among other factors that were considered to be important barriers to a school-based vaccination campaign in the two towns were vaccine side effects (24% in both Gulshan-e-Iqbal and Jamshed), and lack of awareness about the vaccine (34% in Gulshan-e-Iqbal and 38% in Jamshed). Almost one-third of the respondents did not select a third choice when considering factors that may promote vaccine uptake but among those who did respond, risk of disease, vaccine side effects, and lack of awareness about the vaccine were considered to be the most important factors that could affect the vaccination campaign (Table 3).

**Table 3:** Factors identified by respondents from school authorities as barriers to a vaccination campaign in schools in two townships in Karachi, Pakistan

	Gulshan (N = 543)						Jamshed (N = 553)					
	**First choice**		**Second choice**		**Third choice**		**First choice**		**Second choice**		**Third choice**	

	**n**	**%**	**n**	**%**	**n**	**%**	**n**	**%**	**N**	**%**	**n**	**%**

No response	13	2	57	10	182	34	6	1	57	10	167	30

Price of vaccine	321	59	87	16	29	5	383	69	61	11	27	5

Risk of disease	38	7	66	12	54	10	30	5	77	14	72	13

Side effects of the vaccine	44	8	132	24	126	23	40	7	133	24	124	22

Unawareness of the vaccine	114	21	182	34	107	20	86	16	205	37	134	24

Vaccination logistics	13	2	19	3	45	8	8	1	20	4	29	5

The respondents were then asked in a similar fashion to comment on factors that may increase student participation in the immunization program (Table 4). In Gulshan-e-Iqbal town, permission from parents was the first choice among 67% of the respondents, followed by distribution of vaccine-related information materials (47%) and involvement of teachers (28%). In Jamshed town, the school administration staff considered distribution of vaccine-related information materials as an important factor (74%), followed by involvement of the City District Government (55%), and involvement of teachers (32%).

**Table 4:** Factors identified by respondents for school participation in a school vaccination program in Karachi, Pakistan†

	Gulshan (N = 543)						Jamshed (N = 553)					
	**First choice**		**Second choice**		**Third choice**		**First choice**		**Second choice**		**Third choice**	

	**n**	**%**	**n**	**%**	**n**	**%**	**n**	**%**	**N**	**%**	**n**	**%**

No response	12	2	38	7	161	30	9	2	57	10	163	29

Permission from parents	363	67	94	17	33	6	408	74	84	15	24	4

Information pamphlets about the vaccine	111	20	254	47	54	10	83	15	302	55	45	8

Involvement of teachers	16	3	92	17	150	28	15	3	60	11	175	32

Duration of vaccination campaign at each school	5	1	17	3	36	7	5	1	11	2	43	8

Involvement of CDGK^$^	36	7	48	9	109	20	33	6	39	7	103	19

^†^The respondents were given the options from the list and they graded based on order of priority ^$^CDGK - City District Government Karachi

The school census activity in two towns of Karachi provided information on vaccine use and vaccine acceptance, in addition to characteristics (e.g., number of schools, fee structure, and student enrolment) of the schools. Our data from school census shows that there is wide variation in characteristics of the educational institutions of Karachi, Pakistan. Our results also show that majority of education services are provided by private sector. Health and education have recently been dominated by private sector in Pakistan [20,21]. The reasons of such transition have been well documented for Pakistan and other developing countries that include quality of service, consumer satisfaction and access. In such a situation, private schools' significant role not only in education but also in health cannot be ignored. Immunization policies in Pakistan therefore should take into account schools' role and private schools in particular to meet global standards of immunization coverage.

Mapping of the schools identified that many schools are located in close proximity to each other on major streets in the study setting. The smaller schools are located in sections of houses of the school owner or administrator. This type of small school may present logistical issues at the time of the vaccination campaign regarding accessibility. The student size at private schools varies from as low as five students at a school to approximately 3,000 students. Such variety in school size also presents a challenge in terms of properly scheduling a vaccination campaign.

Regular health programs offered at the school level were not very common in this study setting. Increasing health awareness in students in schools may be limited due to limited existing resources at the schools. School-based vaccination campaigns may have to adopt strategies that are attractive and innovative in order to create the need and demand for vaccines in schools. This is in line with the global effort to integrate health activities at schools such as FRESH and WASH campaigns [22-24]. Furthermore, based on the census teams' interviews with school administration, participation rates were very low in immunization programs where the vaccine was offered at a price.

The majority of schools were willing to participate in a school-based vaccination campaign but were unsure of the response from parents. Though there are indications from other endemic areas and countries that people are willing to pay for typhoid vaccine [25-27], schools with past experience of charging a fee for vaccines did not believe a school-based vaccination campaign was very promising. In order to address concerns of parents and school administration, it is therefore worthwhile to focus on increasing awareness in the population about the disease, its consequences, and importance of a vaccine. A well-designed social mobilization campaign targeting decision makers would also help increase acceptance of vaccines in schools.

There are several limitations in this school census. First of all, there were refusals from 58 schools, which may have biased the data as they represent 5% of all schools surveyed. Secondly, the school census could not obtain some important information. There were no records or information from schools regarding vaccination coverage for vaccination campaigns carried out at the schools. The procurement system for vaccines with a user fee was also not elucidated during the interviews. Provided that there is a user fee, vaccines may be priced at private market cost or may have little subsidy. As vaccine cost is a burden to many parents, provision of vaccines at subsidized price through schools will increase coverage. Third, as we interviewed the school administrators regarding the parents' willingness to participate, information may not truly reflect parents' point of views. Further, our respondents for interview varied from owner of the school to a teacher incharge. Therefore the responses may have varied by the characteristics of the respondent and their individual association with the establishment. Lastly, this census was conducted to identify the baseline for the pilot school-based typhoid vaccination program and there were no pre-determined scientific consideration for analysis.

## Conclusions

Despite the recent interests in additional platforms for vaccine introduction as stipulated in the WHO-UNICEF Global Immunization Vision and Strategy [28], challenges remain in implementing vaccination outside of the regular immunization programs. The school census in Karachi addressed key information which may be similarly applicable to other countries. We found that health education or providing health facilities to students in the two townships is not part of the curriculum. In a country like Pakistan, where vaccination coverage is low [29], schools can be used to augment immunization services for children in addition to other health activities such as nutrition. Schools can also be used for catch up vaccination initiatives such as Measles or Polio; however, in order to accomplish this, government's departments of education and health have to collaborate and a joint approach is needed to overcome the problem of low immunization coverage. In order to do that, understanding of school infrastructure becomes important. Schools can be used as important platform for health related initiatives such as legislation of presenting immunization cards at the time of school admission and thereby influence parents' approaches to vaccination. This could also improve retention of immunization cards and can influence vaccination coverage as drop outs are high even for those children who do come in contact with immunization centers [30,31].

The findings from this census have significant implications to developing countries where vaccination coverage is low. Schools could be used as a venue for vaccination programs as has been done in other countries provided that: a) appropriate information is disseminated to parents through a well-designed social mobilization campaign; b) the government include school administration in planning such activities; c) vaccine safety concerns are addressed through a proper adverse events monitoring platform; and d) a multi-sectorial approach is adopted by the Department of Health among the major stakeholders such as private health care providers, religious and community leaders, private school associations, and school administrators.

## Competing interests

The authors declare that they have no competing interests.

## Authors' contributions

SBS - conception and design, interpretation of data, drafting the manuscript. I-uH - acquisition of data, data analysis, manuscript revision. MIK - conception and design, analysis and interpretation of data and manuscript revision. MB - acquisition of data, data analysis, manuscript revision. MM - acquisition of data, data analysis, manuscript revision. IH - acquisition of data, data analysis, manuscript revision. ZS - acquisition of data, data analysis, manuscript revision. AK - data analysis and interpretation, manuscript revision. ARL - acquisition of data, data analysis, manuscript revision. JC - conception and design, manuscript revision critically for intellectual content. ZAB - conception and design, manuscript revision critically for intellectual content. MF - conception and design, and manuscript revision. RLO - conception and design, analysis and interpretation of data and manuscript revision. All authors read and approved the final manuscript.

## Endnotes

^a ^Vi-based Vaccines for Asia (VIVA) Initiative is a five-year program supported by the Bill & Melinda Gates Foundation and coordinated by the International Vaccine Institute (IVI). VIVA's goal is to reduce typhoid fever mortality and morbidity in infants and children in countries that are afflicted by typhoid through the accelerating adoption of existing typhoid vaccines (Vi polysaccharide vaccine), and by developing a new and affordable Vi conjugate vaccines. http://viva.ivi.int

^b ^1USD = 80.69 PKR
